# Supplementary material for: End Sequence Analysis Toolkit (ESAT) expands the extractable information from single-cell RNA-seq data
Source: Genome Res. 2016 Oct;26(10):1397–410. doi: 10.1101/gr.207902.116 (PMC5052061; doi:10.1101/gr.207902.116)
Supplement: Supplemental Material [file supp_gr.207902.116_Supp_Materials.tar.gz › ESAT-esat_v0.1/doc/html/classbroad_1_1core_1_1siphy_1_1_evolutionary_model_parameters.html]

ESAT: broad.core.siphy.EvolutionaryModelParameters Class Reference


ESAT


- Main Page
- Related Pages
- Packages
- Classes
- Files

- Class List
- Class Index
- Class Hierarchy
- Class Members

All Classes Namespaces Files Functions Variables Enumerator Pages

- broad
- core
- siphy
- EvolutionaryModelParameters

Public Member Functions |
List of all members

broad.core.siphy.EvolutionaryModelParameters Class Reference

|  |  |
| --- | --- |
| Public Member Functions | |
|  | EvolutionaryModelParameters (Phylogeny tree, double kappa, double mu, Matrix Q, Matrix pi) |
|  | |
|  | EvolutionaryModelParameters (File sourceFile) throws IOException, ParseException |
|  | |
| EvolutionaryModelParameters | copy () |
|  | |
|  | EvolutionaryModelParameters (double kappa, double[] bg, double mu) |
|  | |
| void | generateHKYQMatrix (double kappa, double scalingFactor) |
|  | |
| void | write (BufferedWriter bw) throws IOException |
|  | |
| String | toString () |
|  | |
| double[] | getBackgroundNucleotideFreqs () |
|  | |
| Matrix | getRateMatrix () |
|  | |
| Phylogeny | getTree () |
|  | |
| void | setTree (Phylogeny tree) |
|  | |
| void | setRateMatrix (Matrix Q) |
|  | |
| void | setBackgroundNucleotideFreqs (Matrix dist) |
|  | |

## Constructor & Destructor Documentation

|  |  |  |  |
| --- | --- | --- | --- |
| broad.core.siphy.EvolutionaryModelParameters.EvolutionaryModelParameters | ( | Phylogeny | *tree*, |
|  |  | double | *kappa*, |
|  |  | double | *mu*, |
|  |  | Matrix | *Q*, |
|  |  | Matrix | *pi* |
|  | ) |  |  |

Here is the caller graph for this function:

|  |  |  |  |  |  |
| --- | --- | --- | --- | --- | --- |
| broad.core.siphy.EvolutionaryModelParameters.EvolutionaryModelParameters | ( | File | *sourceFile* | ) | throws IOException, ParseException |

Here is the call graph for this function:

|  |  |  |  |
| --- | --- | --- | --- |
| broad.core.siphy.EvolutionaryModelParameters.EvolutionaryModelParameters | ( | double | *kappa*, |
|  |  | double[] | *bg*, |
|  |  | double | *mu* |
|  | ) |  |  |

## Member Function Documentation

|  |  |  |  |  |
| --- | --- | --- | --- | --- |
| EvolutionaryModelParameters broad.core.siphy.EvolutionaryModelParameters.copy | ( |  | ) |  |

Here is the call graph for this function:

Here is the caller graph for this function:

|  |  |  |  |
| --- | --- | --- | --- |
| void broad.core.siphy.EvolutionaryModelParameters.generateHKYQMatrix | ( | double | *kappa*, |
|  |  | double | *scalingFactor* |
|  | ) |  |  |

|  |  |  |  |  |
| --- | --- | --- | --- | --- |
| double [] broad.core.siphy.EvolutionaryModelParameters.getBackgroundNucleotideFreqs | ( |  | ) |  |

Here is the caller graph for this function:

|  |  |  |  |  |
| --- | --- | --- | --- | --- |
| Matrix broad.core.siphy.EvolutionaryModelParameters.getRateMatrix | ( |  | ) |  |

|  |  |  |  |  |
| --- | --- | --- | --- | --- |
| Phylogeny broad.core.siphy.EvolutionaryModelParameters.getTree | ( |  | ) |  |

|  |  |  |  |  |  |
| --- | --- | --- | --- | --- | --- |
| void broad.core.siphy.EvolutionaryModelParameters.setBackgroundNucleotideFreqs | ( | Matrix | *dist* | ) |  |

|  |  |  |  |  |  |
| --- | --- | --- | --- | --- | --- |
| void broad.core.siphy.EvolutionaryModelParameters.setRateMatrix | ( | Matrix | *Q* | ) |  |

|  |  |  |  |  |  |
| --- | --- | --- | --- | --- | --- |
| void broad.core.siphy.EvolutionaryModelParameters.setTree | ( | Phylogeny | *tree* | ) |  |

|  |  |  |  |  |
| --- | --- | --- | --- | --- |
| String broad.core.siphy.EvolutionaryModelParameters.toString | ( |  | ) |  |

Here is the call graph for this function:

|  |  |  |  |  |  |
| --- | --- | --- | --- | --- | --- |
| void broad.core.siphy.EvolutionaryModelParameters.write | ( | BufferedWriter | *bw* | ) | throws IOException |

Here is the caller graph for this function:

---

The documentation for this class was generated from the following file:

- java/broad/core/siphy/EvolutionaryModelParameters.java


---

Generated on Fri Aug 15 2014 15:10:57 for ESAT by  

 1.8.7
